# Supplementary material for: IQRray, a new method for Affymetrix microarray quality control, and the homologous organ conservation score, a new benchmark method for quality control metrics
Source: Bioinformatics. 2014 Jan 21;30(10):1392–9. doi: 10.1093/bioinformatics/btu027 (PMC4016700; doi:10.1093/bioinformatics/btu027)
Supplement: Supplementary Data [file supp_30_10_1392__index.html]

IQRray, a new method for Affymetrix microarray quality control, and the homologous organ conservation score, a new benchmark method for quality control metrics — IQRray, a new method for Affymetrix microarray quality control, and the homologous organ conservation score, a new benchmark method for quality control metrics — IQRray, a new method for Affymetrix microarray quality control, and the homologous organ conservation score, a new benchmark method for quality control metrics — Supplementary Data 

# IQRray, a new method for Affymetrix microarray quality control, and the homologous organ conservation score, a new benchmark method for quality control metrics

## Supplementary Data

files

**Files in this Data Supplement:**

- Supplementary Data - zip file
